# Emerging Natural Focal Infectious Diseases in Russia: A Medical–Geographical Study

**DOI:** 10.3390/ijerph17218005

**Published:** 2020-10-30

**Authors:** Svetlana Malkhazova, Polina Pestina, Anna Prasolova, Dmitry Orlov

**Affiliations:** Faculty of Geography, Lomonosov Moscow State University, 119991 Moscow, Russia; sveta_geo@mail.ru (S.M.); polechka10@gmail.com (P.P.); prasolova.geo@yandex.ru (A.P.)

**Keywords:** emerging infectious diseases, natural focal diseases, medical-geographical analysis, disease distribution maps, disease risk assessment, Russia

## Abstract

In Russia, as in other countries, the problem of emerging natural focal infectious diseases (EIDs) became more acute toward the end of the 20th century. However, the situation in Russia is unknown to foreign readers, while the prevention and control of these diseases require international collaboration. The aim of the study is to provide a medical–geographical assessment of the distribution of the main natural focal EIDs in Russia, as well as to present the approaches used in the country to create aggregate maps of risk assessment. To consider its current status, we determined the most important natural focal EIDs for Russia (tick-borne encephalitis, ixodid tick-borne borrelioses, hemorrhagic fever with renal syndrome, Crimean–Congo hemorrhagic fever, West Nile fever, Astrakhan spotted fever, leptospiroses, and tularemia) and analyzed the patterns of their epidemic manifestation. As a result, a working classification of such infections and a series of maps showing the current situation of EID morbidity in Russia were created. To design an aggregated risk map, we developed an original mapping methodology and recalculated the model disease incidence by taking data from administrative units and adjusting them for natural geographical boundaries (biomes) for European Russia, and then evaluated the risk of infection for separate model diseases and for a set of them. The highest risk rates are confined to the northwest regions of European Russia, the Cis-Urals and the Volga region, which are naturally related to forest biomes, as well as to the southern steppe regions of the interfluves between the Volga and the Don, and the foothills of the North Caucasus.

## 1. Introduction

In recent decades, the problem of emerging infectious diseases (EIDs) has been identified as one of the major threats to humanity, given the effects of globalization [1,2,3]. In Russia, as in many other countries, this problem became more acute toward the end of the 20th century. Russia, in common with other parts of the world, experienced the emergence of diseases previously unnoticed across the local populations, while some familiar diseases demonstrated an unexpected increase in morbidity and a tendency of expansion of their nosoareals [4]. However, the situation in Russia is unknown to foreign readers, while the prevention and control of these diseases require international collaboration.

Research focused on EIDs in Russia started from the late 20th century after the Crimean–Congo hemorrhagic fever (CCHF) range began to expand toward neighboring regions [5,6,7]. At the same time, in the southern part of European Russia, new epidemic activity of West Nile fever (WNF) was recorded. In recent years WNF, which has been identified in a limited area of Lower Volga since the end of the nineties, has invaded new territories [8]. Over time, the list of important EIDs found in Russia has been joined by infections considered rare or absent in earlier years: human granulocytic anaplasmosis (*Anaplasma phagocytophilum*), human monocytic ehrlichiosis (*Ehrlichia chaffeensis*), Astrakhan spotted fever (ASF, *Rickettsia conorii*), Omsk hemorrhagic fever (*Omsk hemorrhagic fever virus*), etc. Additionally, the foci of some fairly familiar diseases have become more active, e.g., tick-borne encephalitis (TBE), ixodid tick-borne borrelioses, hemorrhagic fever with renal syndrome (HFRS), leptospiroses and tularemia. Therefore, such diseases are also qualified as EIDs.

Zoonoses constitute a considerable part of EIDs [9,10]. They account for no less than 75% of EIDs [11]. The most important representatives of this group are natural focal diseases. Their agents can circulate among hosts and vectors in natural ecosystems (foci) for an indefinite time period without contact with humans. Humans do not form the necessary links in their parasitic systems but can become accidentally infected upon entering a given focus [12,13]. The geography of such diseases is highly influenced by natural factors, because their hosts and vectors are essential elements of landscapes. Currently, medical–geographical research of the distribution of EIDs is a matter of top priority for health authorities.

The aim of the study is the identification of the most acute emerging natural focal diseases presented in Russia and the creation of aggregate maps of risk assessment. The main objectives of the research are (1) to collect statistical data on natural focal EID morbidity in Russia for two decades (1997–2017); (2) to show the place of Russian EIDs in the international classifications; (3) to elaborate some approaches to their geographical assessment and visualization; (4) to visualize the results by a series of choropleth maps in order to identify the regional difference; and (5) to conduct an analysis of the human infection risks related to the major natural focal diseases in European Russia.

## 2. Materials and Methods

The Rospotrebnadzor’s (Russian Federal Service for Surveillance on Consumer Rights Protection and Human Wellbeing) official statistical data (closed to a wide range of users) on natural focal EID morbidity in Russia for two decades (1997–2017) were used in this research. In order to address the main objectives of the medical–geographical study, a literature review was conducted to examine existing classifications of the entire variety of nosoforms and to identify factors affecting their distribution. Based on statistical data on morbidity analysis, we selected eight relevant nosoforms belonging to EIDs with high levels of epidemic activity in recent years (the annual registration of human cases during the study period and the morbidity rate are higher than the national average): ixodid tick-borne borrelioses; TBE; HFRS; tularemia; leptospiroses; CCHF; WNF and ASF. Model diseases were selected based on their morbidity rates, long-term dynamics, changes in the boundaries of their nosoareals or their internal structure, and the occurrence of epidemic outbreaks.

A series of maps of the current spread of the model infections across Russia was developed based on the federal agency Rospotrebnadzor’s statistics. Mapping methodology was tested by the authors as they prepared numerous maps of the morbidity of natural focal diseases in Russia [13,14]. We used administrative divisions (federal subjects) as map units. The maps reflected morbidity rates for 1997–2017, calculated as the mean annual number of cases per 100,000 persons. For the maps showing the spread of TBE and borrelioses, morbidity was recalculated to account for only those parts of the federal subjects where the presence of vectors was recorded. The northern limit of the range of *Ixodes ricinus* and *Ixodes persulcatus* in Russia was taken as a proxy for the northern boundary of the nosoareal [15].

More detailed assessment of the risks was confined to European Russia. The main problem that arises when mapping and undertaking a spatial analysis of natural focal diseases morbidity is that the initial data of medical statistics are collected from administrative divisions, while the limits of natural foci are determined by natural factors. To assess the risks of emergent natural focal diseases in European Russia, we compiled a synthetic map for the set of selected infections showing their morbidity risks from the perspective of large natural areas, i.e., biomes. A biome is defined as a large zonal ecosystem that unites a number of interconnected smaller natural ecosystems, reflecting the interaction of regional biota with the climate and landscape structure of the territory [16]. Since agents, hosts and vectors of natural focal diseases are inherent elements of the biota, and while natural foci, according to their classical conceptualization [15], are ecosystems associated with certain units of the landscape structure, we regarded biome boundaries as an appropriate natural basis for recalculation of morbidity data.

The task of converting statistical data series for the transition from administrative divisions to geographically determined mapping units was accomplished through the use of geoinformation technologies of spatial analysis, namely overlay operations consisting of superimposing two layers, leading to the formation of a graphic composition that takes into account the distribution of spatial objects, their topology and attributes arithmetically and/or logically derived from the values of the original objects’ attributes [17]. In order to achieve the goal, the overlapping (intersection) operation for the borders of the federal subjects and the boundaries of biomes drawn in accordance with the Russian Biomes Map was performed [18].

As a result of the operation, the boundaries of the intersectional mapping units were obtained. The area-weighted average morbidity rate for the set of model infections in 1997–2017 was calculated for each intersectional unit and weighted in accordance with the unit’s share of the total biome’s area. A summarized value within the limits of the particular biome was produced through calculation of the area-weighted average for the respective intersectional units. This allowed us to characterize the morbidity rates across the biomes. An expert evaluation method was used for the further situation analysis in each federal subject of the European Russia, based on the contemporary scientific literature, information on ecology of the main agents and/or vectors and other epidemiological data.

Based on the developed morbidity risk maps for individual nosoforms, the aggregate morbidity risk from the set of diseases was calculated. We applied a scoring method that is often used in Russian medical–geographical research [19]. The weighted average incidence was ranked into three grades using the natural boundaries method, which was then assigned the corresponding level of disease risk: increased, medium and low. We used a scoring system for this purpose: low risk corresponded to 1 point, average to 2 points, high to 3 points. The mapping unit received 0 points if morbidity was absent. The average value for each biome accounting for all selected diseases was calculated. Based on the obtained values, a complex risk map for biomes was developed, within which a color background shows the aggregate level of risk, and the bar graphs reflect the risk of each particular infection.

## 3. Results and Discussion

As previously noted, there is a significant number of nosoforms related to EIDs which need to be classified due to a wide variety of pathogens as well as differences in the ecology of agents and vectors, mechanisms of transmission, severity of disease, etc. In recent decades, various researchers have proposed a number of classifications by different criteria. In our opinion, the most interesting classifications in the Russian literature are those based on the criteria of the novelty of the pathogen or disease and their combination, the means of the infection identification, and the degree of novelty of the pathogen and the disease itself [20,21]. These classifications are fairly complex and represent attempts to take into account the high variety of EID manifestation. However, analysis of the literature showed that most researchers, following the established tradition, divide EIDs into just two large groups: (a) newly emerging or newly detected infections and (b) infections that existed before but recently for various reasons demonstrate a tendency to expand their nosoareals or an increase in their morbidity (re-emerging diseases). In recent years, some researchers have also identified a third group, diseases deliberately introduced (for purposes of bioterrorism, development of biological weapons, etc.) [2,22].

The second group is the most numerous and needs more detailed classification, taking into account the characteristic manifestations of particular diseases. Therefore, we built a working classification in which four groups of diseases were distinguished based on the character of their geographical distribution. The first group corresponds to the first group of the traditionally accepted classification (newly emerging diseases), and the second group (re-emerging diseases) was divided into three smaller groups. Below are some examples of diseases representing each group.

### 3.1. Newly Emerging or Newly Identified Diseases

Astrakhan spotted fever (ASF) is an acute infectious natural focal disease caused by rickettsia *R. conorii subsp. caspiensis* and transmitted by the ixodid tick *Rhipicephalus pumilio*. In Russia, it is registered in the lower reaches of the Volga in Astrakhan oblast and in the Republic of Kalmykia, and outbreaks in Volgograd oblast are also suspected. According to Rospotrebnadzor, ASF is one of the five most common natural focal diseases transmitted by ticks [23]. This disease is a classic example of a completely new infection. Its study began in 1978 when ASF was first discovered in the Astrakhan oblast [24].

Official registration of ASF cases in Russia began in 2013. During 2013–2015, a total of 1199 cases of ASF in humans were detected, with the overwhelming majority of cases registered in the Astrakhan oblast. Morbidity rate indicators increase from year to year.

The main natural factor affecting the spread of ASF is the presence of its vector, tick *Rh. pumilio*, which makes up the bulk of ixodofauna of Lower Volga region. The main hosts of this tick are wild desert rodents as well as domestic and homeless dogs. Socio-demographic factors, like the size of the population and its settlement patterns, play an important role. The population concentration in the Volga-Akhtuba floodplain and the Volga delta, coupled with the presence of vectors and reservoir animals, leads to the highest morbidity rates in floodplain landscapes. In recent decades, there has been an increase in morbidity in the Volga delta as well as in the desert areas of the river’s left bank, i.e., beyond the areas in which conditions, as previously thought, were the most suitable for existence of a vector. This phenomenon is associated with both the tick’s high environmental plasticity and the expansion of the range of vectors (increase in the number of synanthropic rodents) that occurred as a result of intensive economic use of the territory.

### 3.2. Diseases with Their Areal Limits Static while Changes in Internal Structure and Dynamics Occur

Ixodid tick-borne borrelioses are a group of natural focal infections caused by spirochaetes of the genus *Borrelia* and transmitted by ixodid ticks. Despite their relatively recent discovery, the epidemiology and geographic preconditions of the borrelioses are considered to be fairly well studied. Currently, it is the most common vector-borne disease transmitted by ticks in Russia, Europe and the USA, and a significant part of its global area is located within Russia [25].

**Figure 1:**
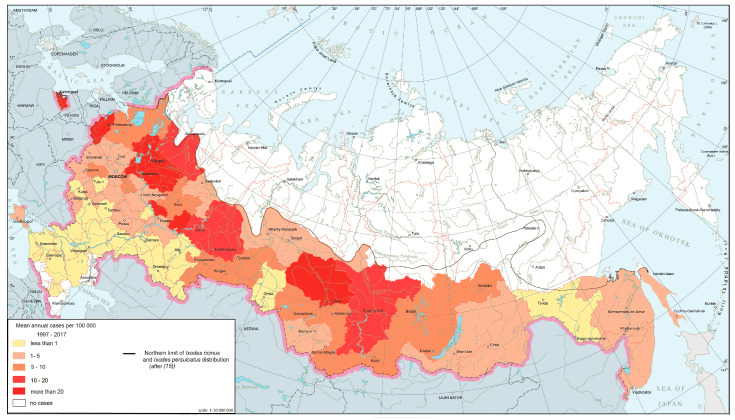
Ixodid tick-borne borrelioses morbidity in Russia, 1997–2017.

The natural foci of borrelioses are confined to the forest zone from the Baltic to Sakhalin, where they are distributed fairly evenly and are characterized by stability. This is reflected in the annual morbidity rates. The nosoform is registered in 74 federal subjects (Figure 1), with the largest number of cases concentrated in 15 regions located in the foothills of the Urals, the middle Urals and the southern parts of Western Siberia, where the risk of infection persists from year to year.

**Figure 2:**
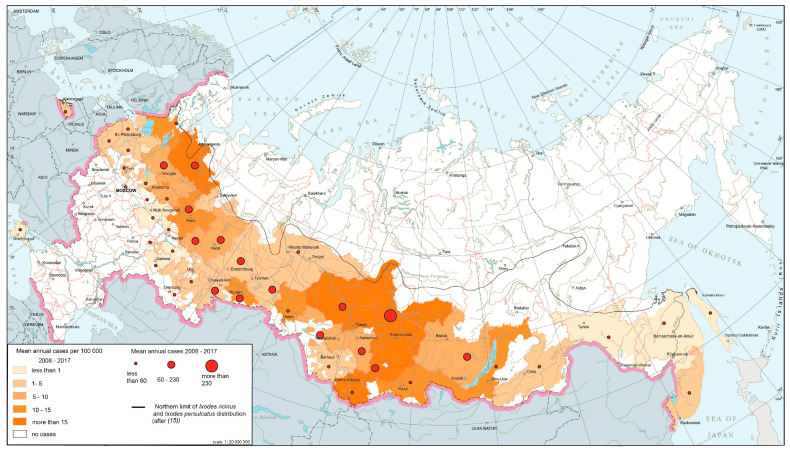
Tick-borne encephalitis morbidity in Russia, 2008–2017.

Tick-borne encephalitis (TBE) is a natural focal disease caused by a polytypic virus [25,26] transmitted by ixodid ticks, primarily *Ixodes persulcatus* and *I. ricinus*. The main part of TBE global area falls within Russian territory. The modern epidemiological situation is characterized by the relative stabilization of morbidity indicators after rapid growth observed in the 1990s, during which corresponding indicators increased ten-fold compared to the late 1980s. At present, TBE is registered in 63 federal subjects (Figure 2). The morbidity varies from 0.01 per 100,000 persons in the Moscow region to more than 60 per 100,000 persons in the southern parts of Western Siberia. The most active foci are confined primarily to the southern parts of Western and Eastern Siberia and the southern Urals.

Borrelioses and TBE are ecologically associated with ixodid ticks, and their nosoareals are limited to the ranges of the main vectors, taiga and castor bean ticks. The taiga tick *I. persulcatus* is confined to various variants of taiga and mixed coniferous forests as well as to mountain taiga massifs. The castor bean tick *I. ricinus* is common in the moderately hygrophilous and mesophilous biotopes of plain and mountain European forests [9].

Borrelioses and TBE can be classified as EIDs due to the fact that in the recent decades their morbidity rates increased as well as the number of biotopes, where infection is possible, especially due to the ticks’ infiltration into urban spaces such as parks and squares. In other words, the territories of the foci and their internal structure are changing. A salient feature of the actual epidemiology of both the borrelioses and TBE is the increasing morbidity among urban residents, which is connected with the ticks’ penetration into city areas on the one hand, and with active development of suburban areas by city dwellers (cultivation of garden and vegetable plots, recreation, etc.) on the other [27].

### 3.3. Diseases Spreading through Expansion of Their Nosoareals to New Territories

Crimean–Congo hemorrhagic fever (CCHF) is a severe natural focal acute human viral infection transmitted by several species of ixodid ticks. An insignificant portion of its nosoareal is on the territory of Russia. CCHF is confined to certain landscapes, namely semi-desert and steppe zones as well as river valleys, which are mainly used for pasturing cattle. In Russia, the main vector is *Hyalomma marginatum,* which prefers territories with low humidity and good warm supply [28]. Morbidity in endemic areas is seasonal and increases during the agricultural season (June–August). It also depends on climatic factors and on the activity of ticks that attack people. Outbreaks of CCHF occur rarely, and its incidence is sporadic, but it is characterized by periodic upsurges, the causes of which are as yet unclear.

**Figure 3:**
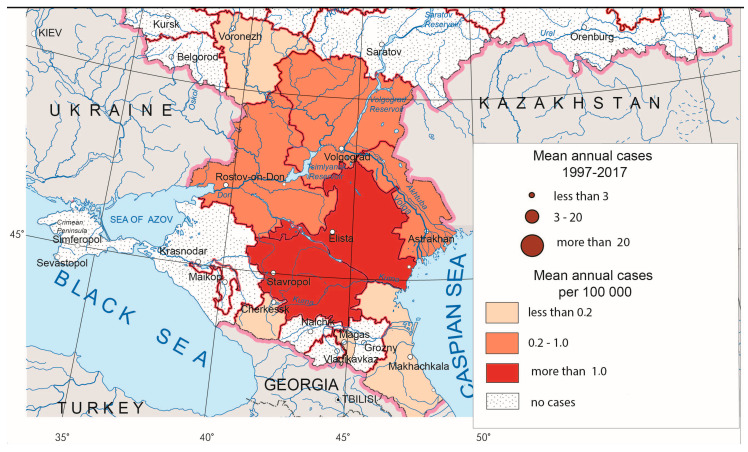
Crimean–Congo hemorrhagic fever morbidity in Russia, 1997–2017.

CCHF foci are situated in the southern parts of European Russia (Figure 3). In recent years, the greatest number of CCHF cases has been observed in Lower Don and the northern Caucasus foothills. Besides activation of the local foci, some time ago, the disease expanded to the north (Lower Volga), to the south (republics of the North Caucasus) and to the east (western Pre-Caspian, where, in recent years, fairly high morbidity rates have been recorded).

West Nile fever (WNF) is an arbovirus natural focal disease, the main hosts of which are birds (mainly belonging to water and near-water complexes and the family *Corvidae*), and its vectors are different species of mosquitoes.

**Figure 4:**
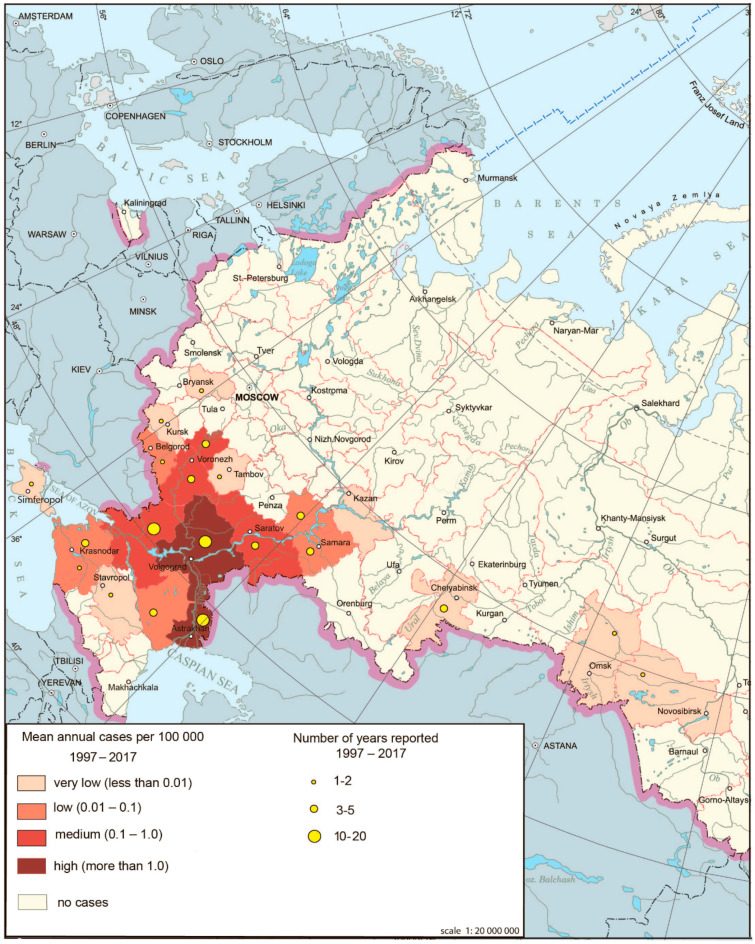
West Nile fever morbidity in Russia, 1997–2017.

The cases of WNF have been registered since 1999 in Russia [29]. Until 2009, this disease was present in five to six federal subjects in the southern parts of European Russia mostly in lower reaches of the Volga and Don Rivers, where it assumed that the foci have existed for a long time. The major outbreak occurred in 2010, affecting not only traditional territories but also new ones. The total number of cases according to Rospotrebnadzor was 448. The area of the disease expanded even more in 2011–2012. In 2012, the disease was registered in 21 federal subjects [30] (Figure 4).

The main natural foci of WNF in Russia are associated with wetlands in the deltas of Volga and Don, areas favorable for migratory birds arriving in the floodplains of these rivers from wintering grounds of countries in which WNF is widely distributed. It was noted that outbreaks in southern Russia are associated primarily with favorable climatic conditions (July temperatures are much higher than +16 °C, which contributes to rapid accumulation of the virus in the salivary glands of mosquitoes) and the presence of hosts and vectors of the virus.

### 3.4. Diseases That Periodically Give Epidemic Outbreaks against the Background of Relatively Stable Mean Morbidity Rates

The most relevant representatives of this group in Russia are serious infections such as tularemia and leptospiroses.

Tularemia is a particularly dangerous infection caused by the highly pathogenic bacterium *Francisella tularensis*. Tularemia natural foci, which are characterized by their robustness, are spread throughout various natural and climatic zones and different types of landscapes in Russia. The most favorable conditions for these foci exist in the forest-steppe and steppe zones, as well as in intrazonal locations, e.g., floodplains and rivers deltas, lake shores and swamp outskirts.

Despite a wide distribution, the mean annual (1997–2017) morbidity rate for tularemia in Russia is fairly low (0.12 per 100,000 persons). However, several large outbreaks of this infection have occurred in Russia over the past 15 years, affecting a significant number of people. More than 300 cases of tularemia were registered in Central Russia in 2005. An even larger outbreak occurred in 2013 in Khanty-Mansiysk Autonomous Okrug, in the center of Western Siberia, with 1005 cases registered (63.9 per 100,000 persons).

This epidemic has become the largest in Russia since the middle of the last century. The most important factors were the favorable conditions for the breeding of mosquitoes belonging to genus *Aedes* coupled with a considerable number of water-voles, the main host of tularemia [31]. Multiple mosquitoes’ bloodsucking cycles during the summer increased the possibility of pathogen transmission from infected rodents to humans, with underlying epizooty among the former. Additional causes of the outbreak consisted of the absence of vaccines against tularemia, low immune stratum, unsatisfactory results of deratization efforts in natural foci, and inefficient implementation of measures to control horn flies [31].

Leptospiroses are also manifested in outbreaks. Their pathogens are transmitted mainly by water via numerous species of moisture-loving rodents, as well as farm animals and dogs. The favorable conditions for survival of these pathogens are abundance of water bodies and a significant number of days in a year with positive temperatures. The leptospiroses outbreaks are often associated with swimming—facts usually explained by non-compliance with the requirements of sanitary and veterinary regulation in terms of safe organization of grazing and watering places, as well as vaccination of animals against leptospiroses.

The leptospiroses nosoareal in Russia covers 74 federal subjects. Notwithstanding the modest morbidity levels, outbreaks occur from time to time. The largest ones were registered on the Black Sea coast of the Caucasus in 1997 with nearly 1500 cases or 29.7 per 100,000 persons, and in the central part of the Russian Plain in 2004 with 700 cases or up to 38.3 per 100,000 persons.

**Figure 5:**
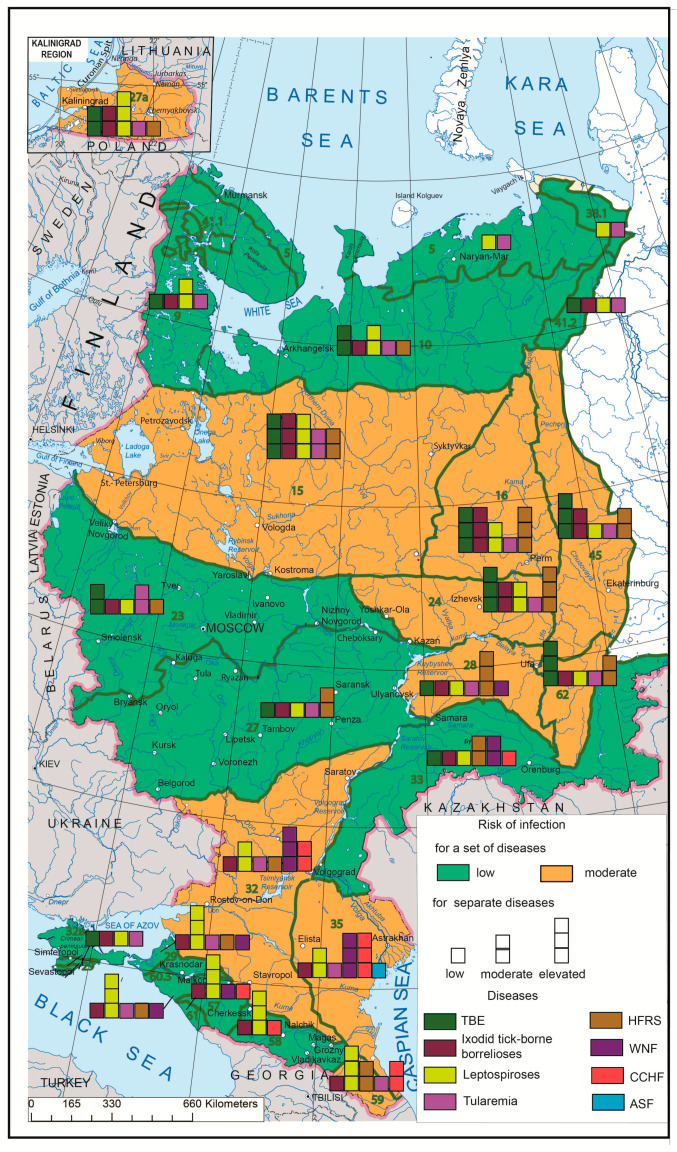
Complex model of emerging infectious disease (EID) risk assessment by biomes. Green lines mark biome boundaries, and green numbers mark biome numbers according to G. Ogureeva [18].

The synthetic map based on a comprehensive analysis of the human infection risks related to the major natural focal diseases in European Russia (Figure 5) shows the uneven distribution of such risks and can serve as a model for assessing the situation throughout Russia. The aggregate risk of morbidity associated with the complex of the aforementioned nosoforms in Russia is not very significant. Its highest rates are confined to the central regions of European Russia, the Urals and the Volga region, which are naturally related to forest biomes, as well as to the southern steppe regions of the interfluves between the Volga and the Don, and the foothills of the North Caucasus. It should be noted that the set of actual infections and their epidemics in these two regions are different, and the risk of infection with certain diseases in some areas is quite high. While the main contributors to morbidity in central Russia are TBE, HFRS and ixodid tick-borne borrelioses, the same role is played by WNF and leptospiroses in the southern parts of European Russia (with CCHF coming first in some regions). From the point of view of the epidemic manifestation of natural focal infections, the most risky are the territories of the Lower Volga, where the largest number of nosoforms is located and where the active centers of WNF and CCHF are situated.

The task of further research is to develop the way of studying environmental change and place it in a healthcare perspective, and the identification of the statistical correspondence between epidemic indicators and geographical factors in the regions of Russia. One of the most commonly cited reasons for EID proliferation in Russia is increased human contact with the natural foci of diseases. Summer cottage development (including garden plots belonging to urban residents where they spend the summer months, which is a very common activity in Russia), enthusiasm for active recreation associated with wildlife visits, and the settlement of new biotopes by vectors (such as urban parks) cause an increase in the morbidity of natural focal infections. To identify such patterns, more detailed studies are needed on the territory of the model regions.

## 4. Conclusions

The aim of this study is to provide a medical–geographical assessment of the distribution of the main natural focal EIDs in Russia for the prevention and control of these diseases on the basis of international collaboration.

Based on the above analysis, we can assume that various emerging and re-emerging infections are present on the territory of the Russian Federation, such as tick-borne encephalitis, ixodid tick-borne borrelioses, hemorrhagic fever with renal syndrome, Crimean–Congo hemorrhagic fever, West Nile fever, Astrakhan spotted fever, leptospiroses, and tularemia. The variability of their manifestations is reflected in the modified classification proposed by authors: (1) newly emerging or newly identified diseases; (2) diseases with their areal limits static while changes in internal structure and dynamics occur; (3) diseases spreading through expansion of their nosoareals to new territories; and (4) diseases that periodically cause epidemic outbreaks against the background of relatively stable mean morbidity rates.

A series of maps of the current spread of the model infections across Russia was developed. The maps show the current situation on the spread of diseases in Russia.

The synthetic map created for the European part of Russia based on the developed methodology shows natural focal EID morbidity risks from the perspective of large natural areas, i.e., biomes. The aggregate risk of morbidity associated with the complex of the aforementioned nosoforms in Russia is not very significant at the present time. However, due to the environmental change, more detailed studies are needed, primarily in regions where the maximum risk of morbidity has been identified (northwest regions of European Russia, the Cis-Urals, the Volga region and the Lower Volga region).
